# “A Quiet Giant in the Fight for Equity”—Hamidah Hussain

**DOI:** 10.3390/tropicalmed8030156

**Published:** 2023-03-03

**Authors:** Aamir Khan, Lubna Samad, Saira Khowaja, Subhash Chandir

**Affiliations:** IRD Global, 1 George Street, Level 10, Singapore 049145, Singapore

**Figure:**
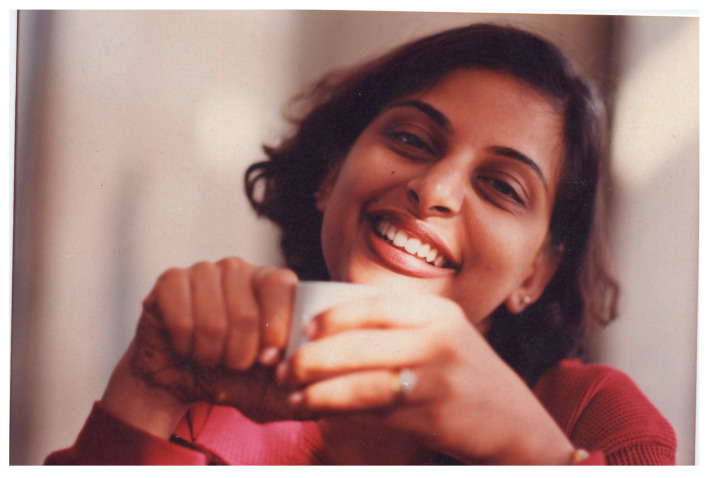


Dr. Hamidah Hussain.

Dr. Hamidah Hussain embodied everything that a global health researcher, advocate, and leader should be. Her work improved and saved countless lives across Pakistan, Bangladesh, Tajikistan, Indonesia, South Africa, and the Philippines. She was the co-founding Director of IRD Global in Singapore, and the founding Country Director for IRD Bangladesh. Her work included active case finding for susceptible and drug-resistant tuberculosis (TB), childhood TB, TB preventive services, vaccine-preventable diseases, and malaria control programs. 

Hamidah previously worked at the Johns Hopkins Bloomberg School of Public Health in Baltimore, BRAC in Dhaka, and Aga Khan University in Karachi. She received her medical degree from Aga Khan University, her Master’s in Health Policy, Planning, and Financing from the London School of Hygiene and Tropical Medicine, and her PhD in health economics from the University of Bergen. She is survived by her husband, Shehzad Noorani, their son Hisbaan, her parents, and a brother. 

She was still raising funds while in hospital for the public health programs that she was passionate about. Hamidah’s extraordinary courage, graciousness, wisdom, generosity, and commitment to the underserved were widely admired. She was a bright star dimmed far too soon, a dedicated force when it came to saving lives. Hamidah will be greatly missed for her contagious smile and contributions to global health. A tribute to her beautiful life can be viewed here (tinyurl.com/tributetohamidah).

## Data Availability

Not applicable.

